# First person – Matthew T. Pereira

**DOI:** 10.1242/dmm.037689

**Published:** 2018-11-28

## Abstract

First Person is a series of interviews with the first authors of a selection of papers published in Disease Models & Mechanisms (DMM), helping early-career researchers promote themselves alongside their papers. Matthew T. Pereira is first author on ‘Effect of dietary additives on intestinal permeability *in vivo* in *Drosophila* and *in vitro* in a human cell co-culture’, published in DMM. Matthew is a 4th year PhD student being paid as a research assistant. The Principal Investigator of his lab is Laura Palanker Musselman, and he is interested in how diet affects physiology.


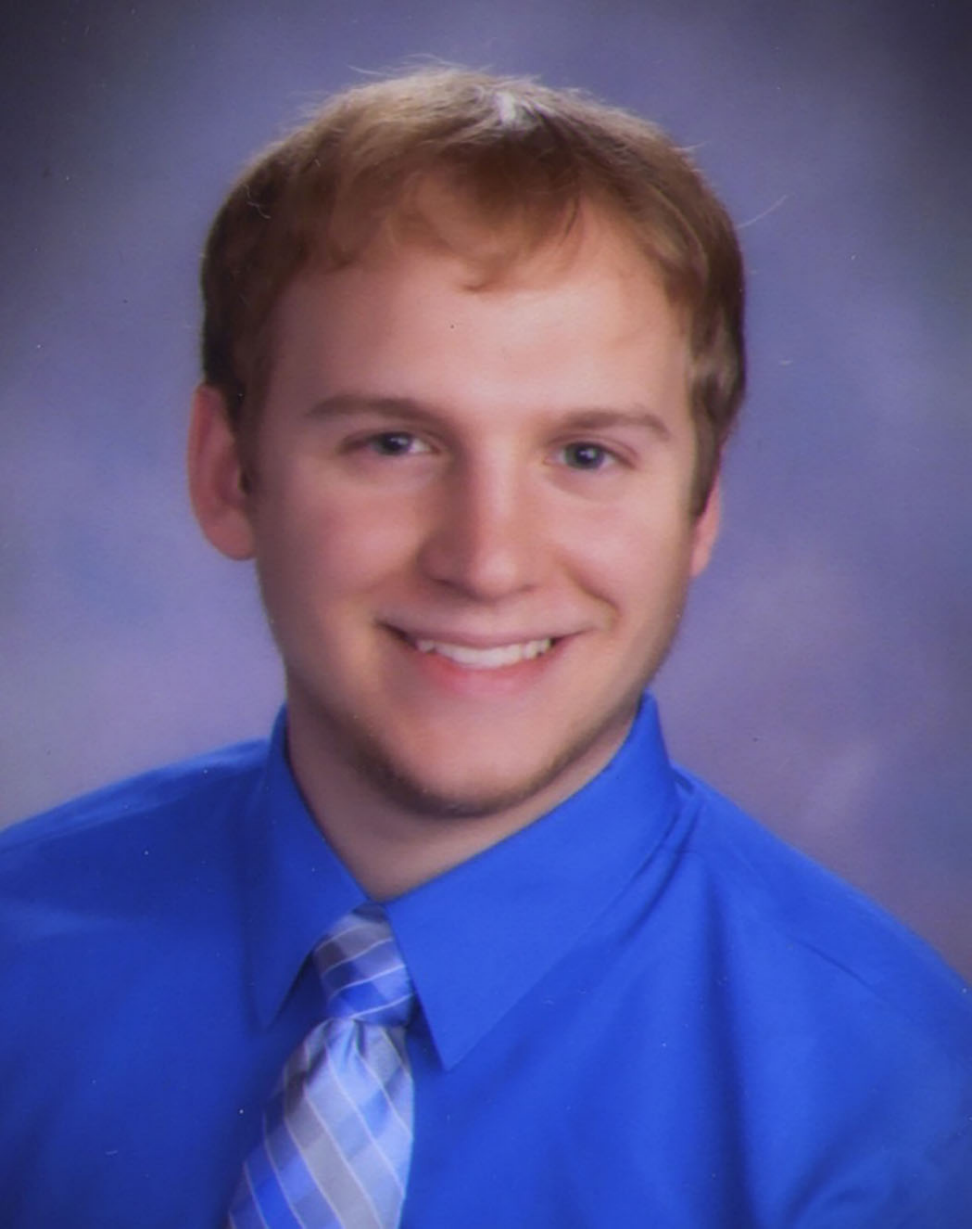


**Matthew T. Pereira**

**How would you explain the main findings of your paper to non-scientific family and friends?**

This study highlights aspects of how detrimental the additives in our food can be to our health. Salt, emulsifiers and added sugar, especially, all have potential to increase the permeability of our gut. Increased gut permeability is associated with aging as well as diseases such as type 2 diabetes, Crohn's disease, celiac disease, multiple sclerosis and irritable bowel syndrome. These rises in permeability may allow harmful microbes to escape the gut into the rest of the body. The aforementioned additives can have an effect on the structure of the gut, the amount of food eaten and overall weight in fruit flies, and we believe this may also affect humans in a similar way.

“Salt, emulsifiers and added sugar, especially, all have potential to increase the permeability of our gut.”

**What are the potential implications of your results for your field of research?**

Our results aid in filling the gap of knowledge surrounding what in our food affects intestinal permeability, as well as highlighting how varying additives can cause different morphological changes in order to do so. These results may encourage others to look at other ways in which additives affect the gut.

**What are the main advantages and drawbacks of the model system you have used as it relates to the disease you are investigating?**

We used both a *Drosophila melanogaster* model as well as a human cell co-culture model. *Drosophila* are very easy to work with, have short lifespans and multiply quickly. On top of this they have very translucent abdomens, allowing you to partially see into their gut. All of these factors make fruit flies a very attractive model to perform the ‘Smurf assay’ on, enabling us to observe the real-time effects diet has on gut permeability *in vivo*, throughout their lifespan. It is very easy to see whether blue-dyed food has leaked from the gut into the peripheral tissues of the fly. The human cell co-culture model allows us to observe a human reaction *in vitro*. Pairing these two models gives us a whole-animal response as well as a human response in which to dually support our findings in two important capacities. As for drawbacks, any long-term survival experiment using flies requires a lot of care. Being that this model can fly, you must be very diligent to not allow any data fly away throughout the duration of the experiment, which could last several months. Human cell co-culture may not fly away on you, but if you do not take the proper precautions you can contaminate your sample and you will have to restart your experiment.

**What has surprised you the most while conducting your research?**

My biggest surprise during this project was seeing how much experimenting with different ‘standard fly diets’ increased gut permeability. From what I read and observed it made sense that altering the diet would alter the degree of which gut permeability was affected, but I assumed the more drastic effects would be from the additives I was studying. I did not expect to see an almost 4-fold increase from essentially lowering the protein content.

**What changes do you think could improve the professional lives of early-career scientists?**

Having an extensive universal online network in which to connect to other scientists, both new and experienced, would help grow and share ideas. An improvement to the ease of networking such as this would promote collaborative studies as well as open doors to researchers who share your field of interest and are able to contribute to or help build your ideas.

**Figure:**
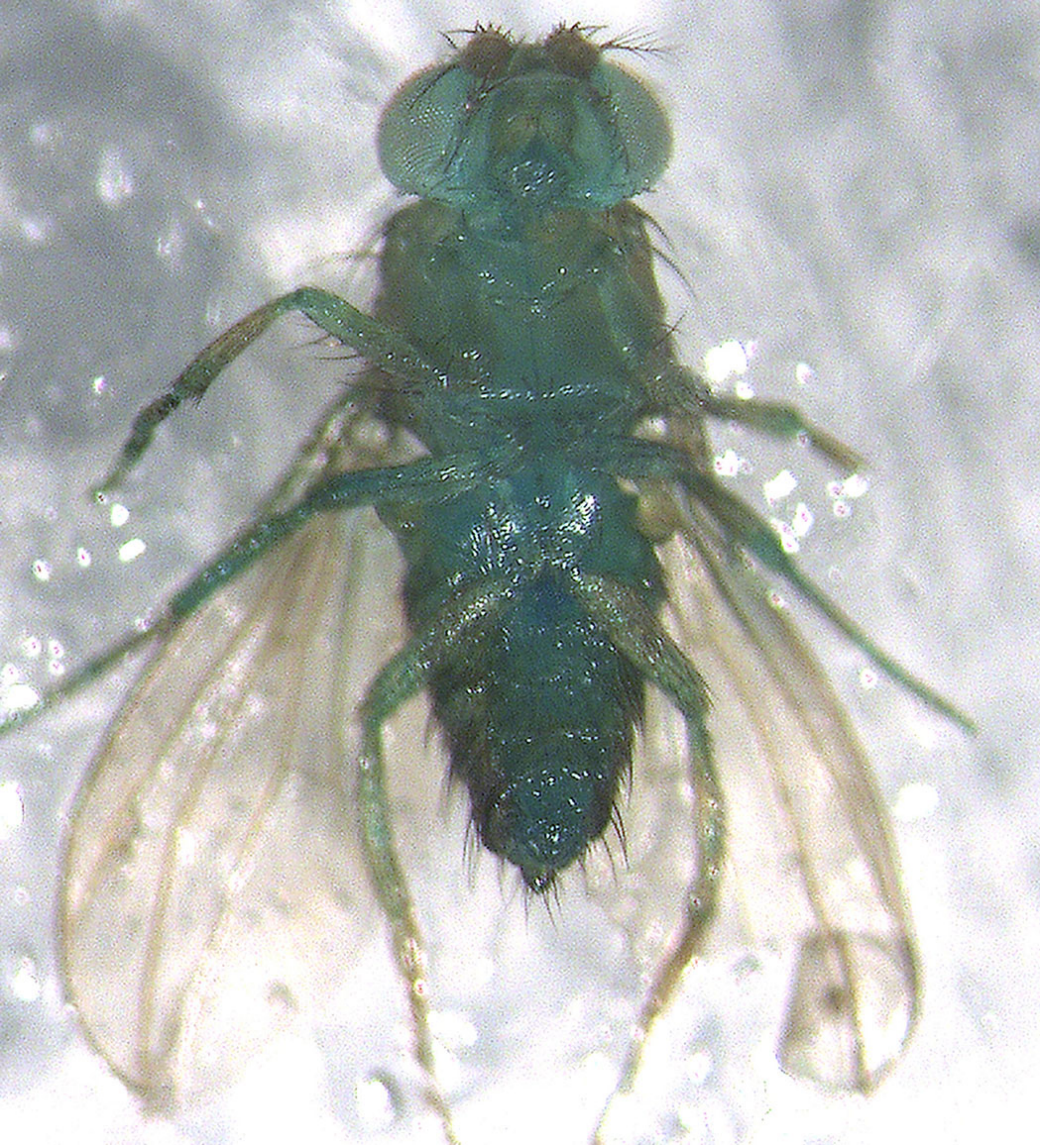
**A ‘Smurfed’ fly in which blue dye has leaked from the gut and entered the peripheral tissues.**

“We were motivated to submit our article to DMM because of DMM's commitment to translational research, and interest in collaborative studies.”

**What 's next for you?**

While working on this project I was also working on another, characterizing a novel protein within the insulin signaling pathway, and I'm just about ready to start writing that as well. Along with finishing this other project I will be researching different options for postdoc positions.

We were motivated to submit our article to DMM because of DMM's commitment to translational research, and interest in collaborative studies. Being that our team of two labs wants to make a difference in the health of each reader's life, we felt DMM was a very good fit for our study.
